# Supplementary material for: Accurate, high-coverage assignment of in vivo protein kinases to phosphosites from in vitro phosphoproteomic specificity data
Source: PLoS Comput Biol. 2022 May 13;18(5):e1010110. doi: 10.1371/journal.pcbi.1010110 (PMC9132282; doi:10.1371/journal.pcbi.1010110)
Supplement: S1 Text — contains the following: Supplemental Methods—Additional details describing data sources, computational methods, and mathematical derivations of specificity models; Fig A—Using relative entropy as a PFM or PSSM column weight provides greater discrimination of columns than using information content; Fig B—Differing theoretical and empirical score ranges in scoring matrix-based models hinders the selection of a universal score cut-off; Fig C—A Venn diagram showing total coverage of the human kinome supported by four kinase-substrate prediction or assignment methods; and Table A—Protein kinases targeted by the chemical inhibitors used in the quantitative phosphoproteomic experiment of Wilkes et al.[41]. (PDF) [file pcbi.1010110.s003.pdf]

Accurate, high-coverage assignment of *in vivo* protein kinases to phosphosites from *in vitro* phosphoproteomic specificity data

Supplemental Materials

Brandon M. Invergo[1,*]

[1]Translational Research Exchange @ Exeter, University of Exeter, Stocker Road, Exeter EX4 4QJ

[*]Correspondence: b.invergo@exeter.ac.uk

Supplemental Methods

Data

In vitro kinase substrates were retrieved from ref. [1], excluding mutant kinases and lipid kinases. Autophosphorylation events were omitted from the analysis. In order to stringently control for false positives, kinase substrates were filtered to include only those that passed both the authors' "SIDIC" and "PTMscore" ($P > 0.75$) criteria [1]. A high-confidence set of human phosphosites was retrieved from ref. [2]. Literature-derived, human phosphosites and kinase-substrate relationships were retrieved from the PhosphoSitePlus database (version 020922) [3] and from ref. [4], and kinase-substrate relationships filtered to remove autophosphorylation.

Kinase physical and indirect interactions were derived from a merged network of interactions from the BioGRID database (version 4.3.194) [5], the BioPlex 293T network (December 2019) [6], and a mass-sepctrometry-based assay of kinase interactions [7]. BioGRID interactions were filtered to exclude biochemical reactions ("Experimental System" = "Biochemical Activity") and genetic interactions. All networks were filtered to exclude self-binding. Protein domains were retrieved from Pfam (version 33.1) [8]. Protein functional association scores were retrieved from the STRING database (version 11.0) [9]. Kinase identities (for IV-KAPhE "substrate is a kinase" feature) and types (S/T or Y) were derived from the *pkinfam.txt* file provided by UniProt and Swiss-Prot (release 2020_02; `https://www.uniprot.org/docs/pkinfam`). For this purpose, dual-specificity kinases were counted as S/T kinases.

Gene Ontology semantic similarity

Semantic similarity of Gene Ontology (GO) terms between a kinase and a putative substrate protein was calculated using the "GOSemSim" package for R (version 2.18.0) [10, 11]. Semantic similarity was calculated using the "Wang" measure and combined using the "BMA" method. Missing values (due to lack of annotation) were imputed from the full *in vivo* feature table via multivariate imputation by chained equations using the "mice" package for R (40 maximum iterations; version 3.13.0) [12].

STRING functional association scores

For all STRING component scores considered ("coexpression", "experimental", "cooccurence", "fusion", and "neighborhood"), values were transformed to be between 0 and 1 by dividing by 1000. Missing values were set to the prior value of 0.041 used by the STRING developers. STRING uses Ensemble identifiers for proteins, whereas the rest of the analysis used Uniprot identifiers. In some cases, many Ensembl identifiers can map to the same Uniprot identifier. In these cases, the means of the scores assigned to all relevant identifiers were used.

Kinase specificity models

Position Frequency Matrix

Sequence windows were first weighted using a position-based sequence-weighting method [13]. Given a set of $n \geq 20$ substrate sequence windows, $\mathbb{S} = \mathbf{S}_1, \mathbf{S}_2, \ldots, \mathbf{S}_i, \ldots, \mathbf{S}_{n-1}, \mathbf{S}_n$, where $\mathbf{S}_i = S_{i1}, S_{i2}, \ldots, S_{i14}, S_{i15}$ and S_{ij} represents the amino acid at position j of sequence i, a weight was assigned to each amino acid a at position j:

$$w(a, j) = \frac{1}{c_j \sum_{i=1}^{n} (S_{ij} = a)} \tag{1}$$

where c_j is the number of unique amino acids found at position j among the substrates in $\mathbb{S}$. Phosphosites near the 5' or 3' tail of the protein may not have 7 residues adjacent to them to form a complete sequence window. At each missing tail position, a 21st amino acid was counted. A weight was calculated for each sequence as the sum of its position-specific residue weights:

$$W(\mathbf{S}_i) = \sum_{j=1}^{15} w(S_{ij}, j) \tag{2}$$

Finally, each sequence's weight was normalized:

$$\widehat{W}(\mathbf{S}_i) = \frac{W(\mathbf{S}_i)}{\sum_{k=1}^{n} W(\mathbf{S}_k)} \tag{3}$$

A 20×15 counts matrix, $\mathbf{r}$ was constructed such that entry r_{aj} contains the weighted count of amino acid a, ignoring missing tails, at position j across the sequences in $\mathbb{S}$:

$$r_{a,j} = n \sum_{i=1}^{n} V(S_{ij}, a) \tag{4}$$

$$V(S_{ij}, a) = \begin{cases} \widehat{W}(\mathbf{S}_i) & \text{if } S_{ij} = a \\ 0 & \text{otherwise} \end{cases} \tag{5}$$

To account for the fact that $\mathbb{S}$ necessarily represents a limited sample of possible substrate sequences, a total number of pseudocounts, B_j, were distributed at position j via 20×15 pseudocount matrix $\mathbf{b}$ as described below. The final PFM, $\mathbf{p}$ was then calculated from the counts and pseudocounts such that the likelihood of observing amino acid a at position j is:

$$PFM_{a,j} = p_{a,j} = \frac{b_{a,j} + r_{a,j}}{B_j + \sum_a r_{a,j}} \tag{6}$$

Following Henikoff and Henikoff [14], a position-specific pseudocount strategy was adopted. For column j the total number of pseudocounts to be distributed among the amino acids is:

$$B_j = T_j + m \times c_j \tag{7}$$

where T_j is the number of missing tails observed in position j among the substrates in $\mathbb{S}$, c_j is defined as in Equation 1, and m is a tune-able parameter, which was fixed at 1 [15]. Note that positions with lower diversity of observed residues will receive fewer pseudocounts.

Pseudocounts were then distributed according to a 20×15 expected frequency matrix $\mathbf{f}$. In the case of PFMs evaluated directly for predictive performance or used in the construction of phosphoproteome-backed PSSMs, $\mathbf{f}$ was constructed as a PFM (as above) on the full set of *in vitro* substrates from all kinases, $\mathbb{S}_{BG}$, with no pseudocounts ($B_j = 0$). In the case of PFMs used in the construction of proteome-backed PSSMs, $\mathbf{f}$ was constructed such that element $f_{a,j} = F_a$, where F_a is the proteomic frequency of residue a. Pseudocount matrix $\mathbf{b}$ was then calculated such that residue a at position j receives $b_{a,j}$ pseudocounts:

$$b_{a,j} = B_j \times f_{a,j} \tag{8}$$

Finally, for each position j, a column weight $\hat{w}_j$ was calculated as the relative entropy (Kullback-Leibler divergence) of the residue frequencies at that position, $\mathbf{p}_j$, versus the background expectation, $\mathbf{f}_j$, rescaled to sum to 15 (the number of positions):

$$w_j = \sum_a p_{a,j} \log_2 \left(\frac{p_{a,j}}{f_{a,j}} \right) \tag{9}$$

$$\hat{w}_j = w_j \times \frac{15}{\sum_j w_j} \tag{10}$$

To assign an unseen substrate, $\mathbf{S}$, to a kinase with PFM $\mathbf{p}$ and column weights $\hat{\mathbf{w}}$, a score is calculated as:

$$s_{PFM}(\mathbf{S}, \mathbf{p}, \hat{\mathbf{w}}) = \prod_j \hat{w}_j p_{S_j,j} \tag{11}$$

Position-Specific Scoring Matrix

20×15 PSSMs were calculated as log-likelihood ratios against the background expectation $\mathbf{f}$:

$$PSSM_{a,j} = \hat{p}_{a,j} = \log_2 \left(\frac{p_{a,j}}{f_{a,j}} \right) \tag{12}$$

As described above, “proteome-backed” PSSMs were calculated against a background expectation of proteomic residue frequencies, while “phosphoproteome-backed” PSSMs were calculated against a position-specific background expectation derived from all phosphosite sequences observed in the experiment.

For assigning an unseen substrate, $\mathbf{S}$, to a kinase with PSSM $\hat{\mathbf{p}}$ and column weights $\hat{\mathbf{w}}$, a score is calculated as:

$$s_{PSSM}(\mathbf{S}, \hat{\mathbf{p}}, \hat{\mathbf{w}}) = \sum_j \hat{w}_j \hat{p}_{S_j,j} \tag{13}$$

For multi-label assignment, a min-max normalized PSSM score, $\hat{s}_{PSSM}$, was calculated to take values between 0 and 1 [16]:

$$a_{min} = \arg\min_a \hat{p}(a, j) \tag{14}$$

$$a_{max} = \arg\max_a \hat{p}(a, j) \tag{15}$$

$$s_{min} = \sum_j \hat{w}_j \hat{p}(a_{min}, j) \tag{16}$$

$$s_{max} = \sum_j \hat{w}_j \hat{p}(a_{max}, j) \tag{17}$$

$$\hat{s}_{PSSM} = \frac{s_{PSSM} - s_{min}}{s_{max} - s_{min}} \tag{18}$$

Multi-label Naïve Bayes

We wish to calculate the posterior probability that a phosphosite is phosphorylated by kinase K, given its surrounding sequence window $\mathbf{S}$. Using Bayes’ Theorem:

$$P(K|\mathbf{S}) = \frac{\mathscr{L}(\mathbf{S}|K)P(K)}{P(\mathbf{S})} \tag{19}$$

$$= \frac{\mathscr{L}(S_1, S_2, \ldots, S_{14}, S_{15}|K)P(K)}{P(\mathbf{S})} \tag{20}$$

By applying the chain rule and then the Naïve Bayes assumption of conditional independence between the positions in **S**, this can be simplified as follows:

$$P(K|\mathbf{S}) = \frac{\mathscr{L}(S_1|S_2, \ldots, S_{15}, K)\mathscr{L}(S_2|S_3, \ldots, S_{15}, K) \ldots \mathscr{L}(S_{14}|S_{15}, K)\mathscr{L}(S_{15}|K)P(K)}{P(\mathbf{S})} \tag{21}$$

$$= \frac{\mathscr{L}(S_1|K)\mathscr{L}(S_2|K) \ldots \mathscr{L}(S_{14}|K)\mathscr{L}(S_{15}|K)P(K)}{P(\mathbf{S})} \tag{22}$$

$$= \frac{P(K)\prod_j \mathscr{L}(S_j|K)}{P(\mathbf{S})} \tag{23}$$

We model the likelihood function, $\mathscr{L}(S_j|K)$ as a categorical distribution. It is then easy to see from Equation 11 that, with the Naïve Bayes assumption of conditional independence:

$$\mathscr{L}(\mathbf{S}|K) = \prod_j \mathscr{L}(S_j|K) = s_{PFM}(\mathbf{S}, \mathbf{p}_K, \hat{\mathbf{w}} = \{1, 1, \ldots, 1, 1\}) \tag{24}$$

By altering the values in $\hat{\mathbf{w}}$, we arrive at a weighted Naïve Bayes formulation:

$$P(K|\mathbf{S}) = \frac{P(K)\prod_j \hat{w}_j \mathscr{L}(S_j|K)}{P(\mathbf{S})} \tag{25}$$

$$= \frac{P(K) s_{PFM}(\mathbf{S}, \mathbf{p}_K, \hat{\mathbf{w}}_K)}{P(\mathbf{S})} \tag{26}$$

So, the conditional likelihood function $\mathscr{L}(\mathbf{S}|K)$ is calculated as described for PFMs.

The prior probability, $P(K)$ was empirically estimated as the proportion of all substrates observed in the experiment ($\mathbb{S}_{BG}$) that were observed as phosphorylated by kinase K ($\mathbb{S}_K$):

$$P(K) = \frac{|\mathbb{S}_K|}{|\mathbb{S}_{BG}|} \tag{27}$$

Typically the marginal probability, $P(\mathbf{S})$, is ignored in Naïve Bayes assignment as it is just a normalizing constant. However, in multi-label Naïve Bayes, normalization is necessary to compare probabilities between classes (kinases) [17]. By the law of total probability:

$$P(\mathbf{S}) = \mathscr{L}(\mathbf{S}|K)P(K) + \mathscr{L}(\mathbf{S}|\bar{K})P(\bar{K}) \tag{28}$$

The second term represents the likelihood of sequence $\mathbf{S}$ being phosphorylated by any other kinase in the experiment ($\bar{K}$). This is computed exactly as for kinase K, using sequences not phosphorylated by K, $\mathbb{S}_{\bar{K}}$, to build the PFM and calculate the prior probability:

$$\mathbb{S}_{\bar{K}} = \mathbb{S}_{BG} - \mathbb{S}_K \tag{29}$$

To perform multi-label assignment of phosphosite sequence $\mathbf{S}$, for each kinase K_i in the set of kinases used in the experiment, $\mathbb{K}$, posterior probabilities were calculated as

$$P(K_i|\mathbf{S}) = \frac{P(K_i)s_{PFM}(\mathbf{S},\mathbf{p}_{K_i},\hat{\mathbf{w}}_{K_i})}{P(K_i)s_{PFM}(\mathbf{S},\mathbf{p}_{K_i},\hat{\mathbf{w}}_{K_i}) + P(\bar{K}_i)s_{PFM}(\mathbf{S},\mathbf{p}_{\bar{K}_i},\hat{\mathbf{w}}_{\bar{K}_i})} \tag{30}$$

$$P(\bar{K}_i|\mathbf{S}) = \frac{P(\bar{K}_i)s_{PFM}(\mathbf{S},\mathbf{p}_{\bar{K}_i},\hat{\mathbf{w}}_{\bar{k}_i})}{P(K_i)s_{PFM}(\mathbf{S},\mathbf{p}_{K_i},\hat{\mathbf{w}}_{K_i}) + P(\bar{K}_i)s_{PFM}(\mathbf{S},\mathbf{p}_{\bar{K}_i},\hat{\mathbf{w}}_{\bar{K}_i})} \tag{31}$$

Kinase K_i was assigned to the phosphosite with sequence $\mathbf{S}$ if $P(K_i|\mathbf{S}) > P(\bar{K}_i|\mathbf{S})$ [17]. This is equivalent to the condition that $P(K_i|\mathbf{S}) > 0.5$, thus $P(\bar{K}_i|\mathbf{S})$ need not be calculated.

Naïve Bayes+

The Naïve Bayes+ model is an extension of the multi-label Naïve Bayes model described above. In addition to the sequence evidence $\mathbf{S}$, additional Bernoulli-distributed features were added – $I_{K,T}$ or $\bar{I}_{K,T}$: presence or absence of a direct physical interaction between kinase K and substrate protein T; $J_{K,T}$ or $\bar{J}_{K,T}$: presence or absence of an indirect physical interaction between them; D or $\bar{D}$: the phosphosite is or is not in a predicted domain on T; and E or $\bar{E}$: substrate T does or does not carry a predicted domain that is enriched among K's substrates or physical interaction partners. An indirect interaction was defined as the substrate protein T being "two hops" away from K on the protein interaction network, i.e. they share a neighbor.

Thus, for example, the Naïve Bayes formulation of the posterior probability that kinase K phosphorylates a site with sequence $\mathbf{S}$, that is not in a domain $\bar{D}$, with no direct physical interaction $\bar{I}_{K,T}$, an indirect interaction $J_{K,T}$, and no enriched domains $\bar{E}$ would be:

$$P(K|\mathbf{S},\bar{D},\bar{I}_{K,T},J_{K,T},\bar{E}) = \frac{\mathscr{L}(\mathbf{S}|K)\mathscr{L}(\bar{D}|K)\mathscr{L}(\bar{I}_{K,T}|K)\mathscr{L}(J_{K,T}|K)\mathscr{L}(\bar{E}|K)P(K)}{P(S)} \tag{32}$$

$$\begin{aligned} P(S) = {} & \mathscr{L}(\mathbf{S}|K)\mathscr{L}(\bar{D}|K)\mathscr{L}(\bar{I}_{K,T}|K)\mathscr{L}(J_{K,T}|K)\mathscr{L}(\bar{E}|K)P(K) + \\ & \mathscr{L}(\mathbf{S}|\bar{K})\mathscr{L}(\bar{D}|\bar{K})\mathscr{L}(\bar{I}_{K,T}|\bar{K})\mathscr{L}(J_{K,T}|\bar{K})\mathscr{L}(\bar{E}|\bar{K})P(\bar{K}) \end{aligned} \tag{33}$$

For all four additional features, the Bernoulli parameter p was estimated by calculating the fraction of K's substrates that have the feature (or, for $\bar{K}$, the fraction of all sites that are not substrates of K). Domains enriched within the substrate proteins or interaction partners of kinase K were identified via Fisher's Hypergeometric Test. For each unique domain predicted to be on at least one substrate protein, presence or absence of that domain among proteins were counted contingent upon the proteins being either substrates or physical interaction partners of K or not. p-values were adjusted for false discovery rate and tested at a critical value of 0.05.

Approximate logistic relationship between phosphoproteome-backed PSSM score and Naïve Bayes posterior probability

As described above, the definition of the Naïve Bayes posterior probability of assigning kinase K to a phosphosite with sequence window $\mathbf{S}$ is:

$$P(K|\mathbf{S}) = \frac{\mathscr{L}(\mathbf{S}|K)P(K)}{P(\mathbf{S})} \tag{34}$$

$$\mathscr{L}(\mathbf{S}|K) = \mathscr{L}(S_1|K)\mathscr{L}(S_2|K)...\mathscr{L}(S_{14}|K)\mathscr{L}(S_{15}|K) \tag{35}$$

In terms of the likelihood, the phosphoproteome-backed PSSM score function $s_{PSSM}(\mathbf{S}, \hat{\mathbf{p}}_K, \hat{\mathbf{w}}_K)$ can be re-written as:

$$s_{PSSM}(\mathbf{S}, \hat{\mathbf{p}}_K, \hat{\mathbf{w}}_K) = \log_2\left(\frac{\mathscr{L}(\mathbf{S}|K)}{\mathscr{L}(\mathbf{S})}\right) \tag{36}$$

Likelihood $\mathscr{L}(\mathbf{S})$ is the overall likelihood of observing a phosphosite with sequence window $\mathbf{S}$, as calculated using the full phosphoproteome background set, $\mathbb{S}_{BG}$. Likelihood $\mathscr{L}(\mathbf{S}|K)$ is the likelihood of observing a phosphosite with sequence window $\mathbf{S}$ among the substrates of kinase K, as calculated using a set of known substrates of kinase K, $\mathbb{S}_K$. Furthermore, we assume:

$$\mathbb{S}_K \subset \mathbb{S}_{BG} \tag{37}$$

$$|\mathbb{S}_K| \ll |\mathbb{S}_{BG}| \tag{38}$$

We apply a logit transformation to the Naïve Bayes posterior probability $P(K|\mathbf{s})$, using 2 as our logarithm base:

$$\begin{aligned}
\text{logit}(P(K|\mathbf{S})) &= \log_2\left(\frac{P(K|\mathbf{S})}{1-P(K|\mathbf{S})}\right) \\
&= \log_2\left(\frac{P(K|\mathbf{S})}{P(\overline{K}|\mathbf{S})}\right) \\
&= \log_2\left(\frac{\mathscr{L}(\mathbf{S}|K)P(K)}{\mathscr{L}(\mathbf{S}|\overline{K})P(\overline{K})}\right) \\
&= \log_2\left(\frac{\mathscr{L}(\mathbf{S}|K)}{\mathscr{L}(\mathbf{S}|\overline{K}))}\right) + \log_2\left(\frac{P(K)}{P(\overline{K})}\right)
\end{aligned} \tag{39}$$

Given that $|\mathbb{S}_K| \ll |\mathbb{S}_{BG}|$ (Equation 38), $\mathscr{L}(\mathbf{S}|\overline{K}) \approx \mathscr{L}(\mathbf{S})$. Put simply, the likelihood model constructed from a set of over 14000 phosphosites will not be strongly impacted if one were first to omit a subset of a few hundred sites on average. As a result:

$$\begin{aligned}
\text{logit}(P(K|\mathbf{S})) &\approx \log_2\left(\frac{\mathscr{L}(\mathbf{S}|K)}{\mathscr{L}(\mathbf{S}))}\right) + \log_2\left(\frac{P(K)}{P(\bar{K})}\right) \\
\text{logit}(P(K|\mathbf{S})) &\approx s_{PSSM}(\mathbf{S}, \hat{\mathbf{p}}_K, \hat{\mathbf{w}}_K) + \log_2\left(\frac{P(K)}{P(\bar{K})}\right) \\
s_{PSSM}(\mathbf{S}, \hat{\mathbf{p}}_K, \hat{\mathbf{w}}_K) &\approx \text{logit}(P(K|\mathbf{S})) - \log_2\left(\frac{P(K)}{P(\bar{K})}\right)
\end{aligned} \tag{40}$$

From Equation 38, it also follows that $|\mathbb{S}_K| < |\mathbb{S}_{\bar{K}}|$, so $P(K) < P(\bar{K})$ and $\log_2\left(\frac{P(K)}{P(\bar{K})}\right) < 0$. This imposes a dependence of the relationship on $|\mathbb{S}_K|$: a larger $|\mathbb{S}_K|$ causes a lower inflection point in the inverse logistic fit, i.e. the PSSM score $s_{PSSM}(\mathbf{S}, \hat{\mathbf{p}}_K, \hat{\mathbf{w}}_K)$ at which $P(K|\mathbf{S}) = 0.5$ and $\text{logit}(P(K|\mathbf{S})) = 0$.

Supplemental Figures

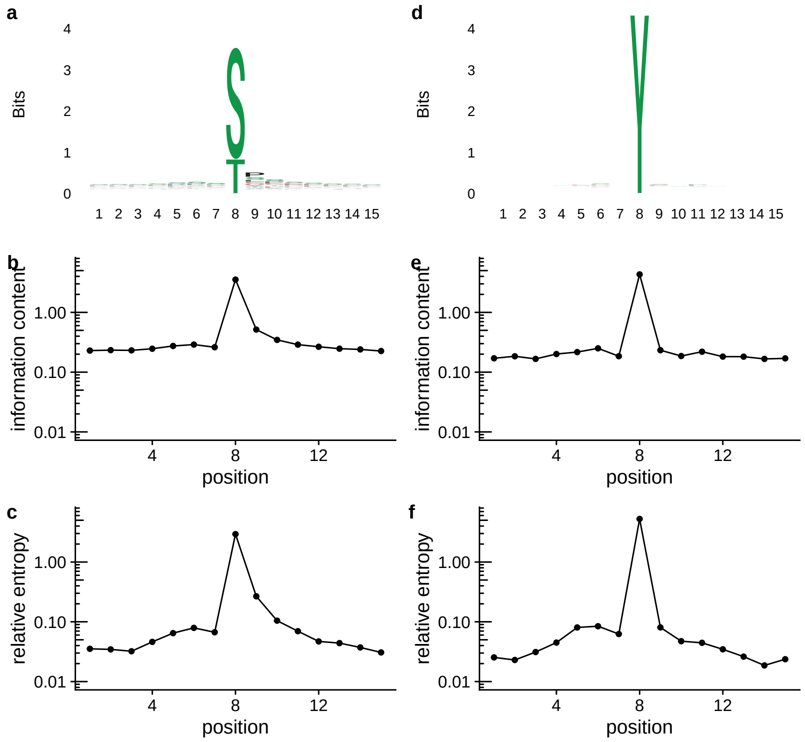

Fig A: **Using relative entropy as a PFM or PSSM column weight provides greater discrimination of columns than using information content.** a) Sequence logo of all S/T sites in a high-confidence human phosphoproteome [2]. b) Information content at each position among S/T sites. c) Relative entropy versus proteomic residue frequencies at each position among S/T sites. Relative entropy provides greater separation between positions. d-f) As in (a-c) for Y sites.

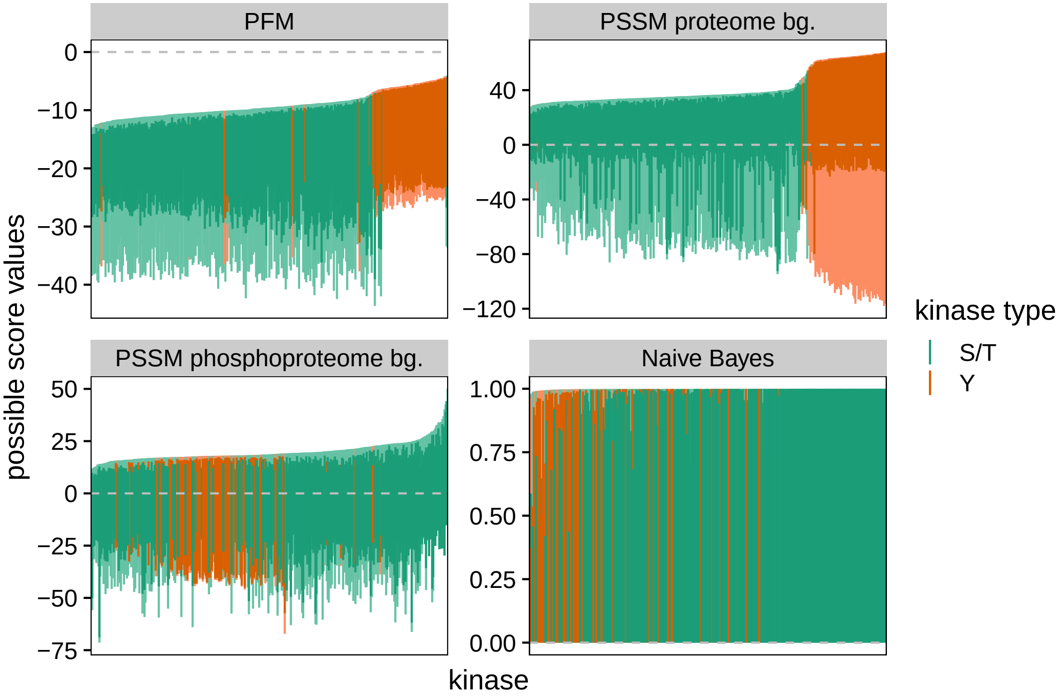

Fig B: **Differing theoretical and empirical score ranges in scoring matrix-based models hinders the selection of a universal score cut-off.** Each vertical bar represents one kinase, colored by S/T (green) or Y kinases (orange). Light bars show theoretical score ranges and dark bars show empirical score ranges for the four sequence-only kinase specificity models. Empirical ranges were identified using the high-confidence human phosphoproteome [2]. PFM scores are shown $\log_{10}$-transformed. Note that PFM and PSSM-based models have very different ranges between kinases. The maximum theoretical range also varies, making universal cutoff definitions difficult. Also, PFMs and PSSMs do not utilize their full range for all kinases when scoring physiologically occurring phosphosites. The Naïve Bayes model exhibits none of these problems.

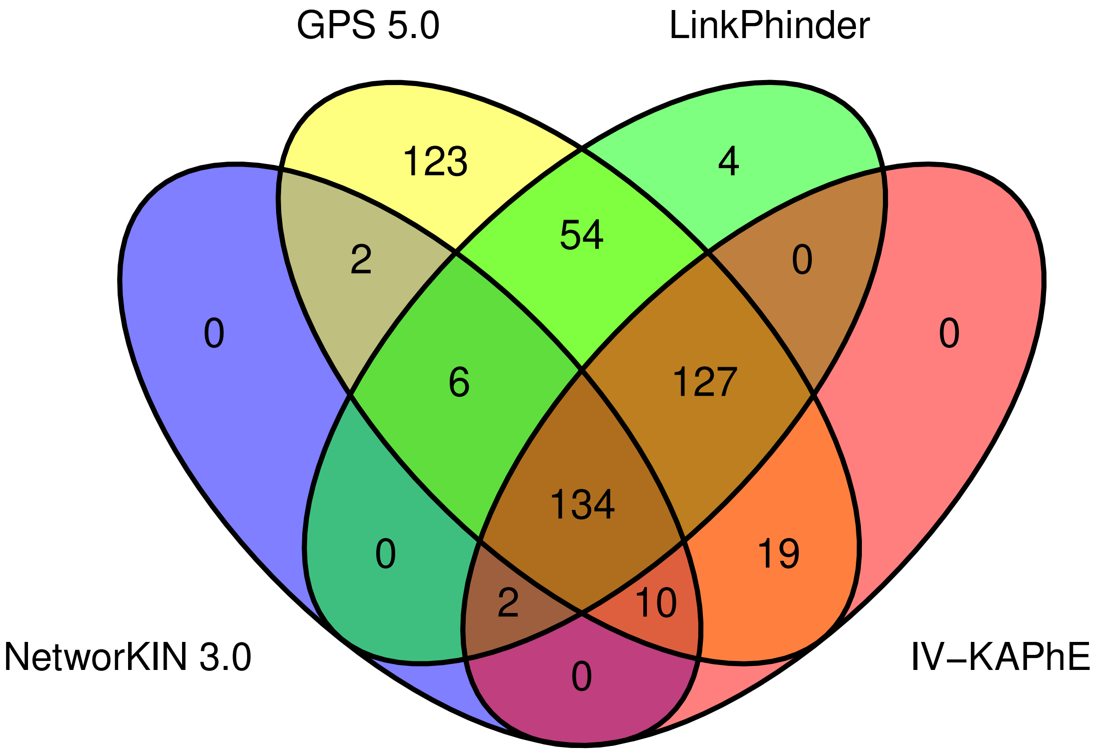

Fig C: **A Venn diagram showing total coverage of the human kinome supported by four kinase-substrate prediction or assignment methods.** Values reflect numbers of kinases. GPS 5.0 has the largest coverage, while NetworKIN 3.0's coverage is significantly lower than the others. IV-KAPhE does not cover any kinases not covered by the other methods.

Supplemental Tables

Table A: Protein kinases targeted by the chemical inhibitors used in the quantitative phosphoproteomic experiment of Wilkes *et al.* [18].

Condition Name	Chemical Name	Target Kinases (gene symbol)	Target Information Source
AKT[1]	Akt inhibitor VIII	AKT1 AKT2 AKT3	https://www.selleckchem.com/products/akti-1-2.html
AKT[2]	MK-2206	AKT1 AKT2 AKT3	https://www.selleckchem.com/products/MK-2206.html
CAMK[1]	KN-93	CAMK2A CAMK2B CAMK2D CAMK2G	https://www.selleckchem.com/products/kn-93.html
CAMK[2]	KN-62	CAMK2A CAMK2B CAMK2D CAMK2G CAMK1 CAMK1D CAMK1G CAMK4	https://www.selleckchem.com/products/kn-62.html
EGFR[1]	PD-168393	EGFR	https://www.selleckchem.com/products/pd168393.html
EGFR[2]	PD-153035	EGFR	https://www.selleckchem.com/products/pd153035.html
ERK[1]	ERK inhibitor	MAPK1 MAPK3	https://www.sigmaaldrich.com/catalog/product/mm/328007
ERK[2]	ERK inhibitor II	MAPK1 MAPK3	https://www.sigmaaldrich.com/catalog/product/mm/328006m
MEK[1]	GSK-1120212	MAP2K1 MAP2K2	https://www.selleckchem.com/products/gsk1120212-jtp-74057.html
MEK[2]	U0126	MAP2K1 MAP2K2	https://www.medchemexpress.com/u-0126.html
MTOR[1]	KU-0063794	MTOR	https://pubchem.ncbi.nlm.nih.gov/compound/Ku-0063794#section=Biomolecular-Interactions-and-Pathways
MTOR[2]	Torin-1	MTOR	https://www.cellsignal.com/products/activators-inhibitors/torin-1/14379
P70S6K[1]	PF-4708671	RPS6KB1	https://www.selleckchem.com/products/pf-4708671.html
P70S6K[2]	DG2	RPS6KB1	https://www.sigmaaldrich.com/catalog/product/mm/559274

PI3K[1]	GDC-0941	PIK3CA PIK3CB PIK3CD PIK3CG	https://www.stemcell.com/gdc-0941.html
PI3K[2]	PI-103	PIK3CA PIK3CB PIK3CD PIK3CG MTOR	https://www.stemcell.com/gdc-0941.html
PKC[1]	Gö-6976	PRKCA PRKCB PRKCG PRKCD PRKCZ JAK2 FLT3	https://www.selleckchem.com/products/go6976.html https://pubmed.ncbi.nlm.nih.gov/16956345/
PKC[2]	BIM-1	PRKCA PRKCB PRKCG	https://www.selleckchem.com/products/gf109203x.html
ROCK[1]	H-1152 dihydrochloride	ROCK2 CAMK2A CAMK2B CAMK2D CAMK2G PRKG1 PRKG2 AURKA PRKACA PRKACB PRKACG PRKCA PRKCB PRKCB PRKCG PRKCD PRKCE PRKCH PRKCQ PRKCI PRKCZ	https://www.selleckchem.com/products/h-1152-dihydrochloride.html
ROCK[2]	Y-27632	ROCK1 ROCK2	https://www.selleckchem.com/products/Y-27632.html

References

[1] Sugiyama N, Imamura H, Ishihama Y. Large-Scale Discovery of Substrates of the Human Kinome. Scientific Reports. 2019;9(1):10503. doi:10.1038/s41598-019-46385-4.

[2] Ochoa D, Jarnuczak AF, Viéitez C, Gehre M, Soucheray M, Mateus A, et al. The Functional Landscape of the Human Phosphoproteome. Nat Biotechnol. 2020;38(3):365–373. doi:10.1038/s41587-019-0344-3.

[3] Hornbeck PV, Zhang B, Murray B, Kornhauser JM, Latham V, Skrzypek E. PhosphoSitePlus, 2014: Mutations, PTMs and Recalibrations. Nucleic Acids Research. 2015;43(Database–issue):D512–20. doi:10.1093/nar/gku1267.

[4] Bachman JA, Gyori BM, Sorger PK. Assembling a Phosphoproteomic Knowledge Base Using ProtMapper to Normalize Phosphosite Information from Databases and Text Mining. bioRxiv. 2019; p. 822668. doi:10.1101/822668.

[5] Oughtred R, Rust J, Chang C, Breitkreutz BJ, Stark C, Willems A, et al. The BioGRID Database: A Comprehensive Biomedical Resource of Curated Protein, Genetic, and Chemical Interactions. Protein Sci. 2021;30(1):187–200. doi:10.1002/pro.3978.

[6] Huttlin EL, Bruckner RJ, Navarrete-Perea J, Cannon JR, Baltier K, Gebreab F, et al. Dual Proteome-Scale Networks Reveal Cell-Specific Remodeling of the Human Interactome. Cell. 2021;184(11):3022–3040.e28. doi:10.1016/j.cell.2021.04.011.

[7] Buljan M, Ciuffa R, van Drogen A, Vichalkovski A, Mehnert M, Rosenberger G, et al. Kinase Interaction Network Expands Functional and Disease Roles of Human Kinases. Mol Cell. 2020;79(3):504–520.e9. doi:10.1016/j.molcel.2020.07.001.

[8] Finn RD, Coggill P, Eberhardt RY, Eddy SR, Mistry J, Mitchell AL, et al. The Pfam Protein Families Database: Towards a More Sustainable Future. Nucleic Acids Research. 2016;44(D1):D279–85. doi:10.1093/nar/gkv1344.

[9] Szklarczyk D, Gable AL, Lyon D, Junge A, Wyder S, Huerta-Cepas J, et al. STRING V11: Protein-Protein Association Networks with Increased Coverage, Supporting Functional Discovery in Genome-Wide Experimental Datasets. Nucleic Acids Res. 2019;47(D1):D607–D613. doi:10.1093/nar/gky1131.

[10] Yu G, Li F, Qin Y, Bo X, Wu Y, Wang S. GOSemSim: An R Package for Measuring Semantic Similarity among GO Terms and Gene Products. Bioinformatics. 2010;26(7):976–978. doi:10.1093/bioinformatics/btq064.

[11] Yu G. Gene Ontology Semantic Similarity Analysis Using GOSemSim. In: Kidder BL, editor. Stem Cell Transcriptional Networks: Methods and Protocols. Methods in Molecular Biology. New York, NY: Springer US; 2020. p. 207–215.

[12] van Buuren S, Groothuis-Oudshoorn K. Mice: Multivariate Imputation by Chained Equations in R. Journal of Statistical Software. 2011;45(1):1–67. doi:10.18637/jss.v045.i03.

[13] Henikoff S, Henikoff JG. Position-Based Sequence Weights. Journal of Molecular Biology. 1994;243(4):574–578.

[14] Henikoff JG, Henikoff S. Using Substitution Probabilities to Improve Position-Specific Scoring Matrices. Comput Appl Biosci. 1996;12(2):135–143.

[15] Invergo BM, Petursson B, Akhtar N, Bradley D, Giudice G, Hijazi M, et al. Prediction of Signed Protein Kinase Regulatory Circuits. Cell Syst. 2020;10(5):384–396.e9. doi:10.1016/j.cels.2020.04.005.

[16] Wagih O, Reimand J, Bader GD. MIMP: Predicting the Impact of Mutations on Kinase-Substrate Phosphorylation. Nat Methods. 2015;12(6):531–533. doi:10.1038/nmeth.3396.

[17] Zhang ML, Peña JM, Robles V. Feature Selection for Multi-Label Naive Bayes Classification. Information Sciences. 2009;179(19):3218–3229. doi:10.1016/j.ins.2009.06.010.

[18] Wilkes EH, Terfve C, Gribben JG, Saez-Rodriguez J, Cutillas PR. Empirical Inference of Circuitry and Plasticity in a Kinase Signaling Network. Proc Natl Acad Sci USA. 2015;112(25):7719–7724. doi:10.1073/pnas.1423344112.
